# Stroke in postpartum: a case report on rare presentation of primary Sjögren’s syndrome

**DOI:** 10.1097/MS9.0000000000005733

**Published:** 2026-10-12

**Authors:** Sabal Pandey, Ashim Batajoo

**Affiliations:** a Medicine Department, Chautara Hospital, Sindhupalchok, Bagmati Province, Nepal; b Medicine Department, Manipal College of Medical Sciences, Pokhara, Gandaki Province, Nepal

**Keywords:** case report, postpartum, postpartum flare of a rheumatological condition, primary Sjögren’s syndrome, stroke

## Abstract

**Introduction:**

Sjögren’s syndrome is a systemic disease that can affect all organs. Postpartum stroke is a rare presentation of Sjögren’s syndrome, requiring a multidisciplinary approach.

**Case:**

A 10-day postpartum 28-year-old woman presented with an acute onset of right-sided weakness with deviation of the mouth to the left. She also complained of dryness of the eyes, generalized fatigability, and intermittent generalized aches and pains. On examination, power in the right upper and lower limbs was 1/5, with a right upper motor neuron-type facial palsy and a positive Babinski sign. Non-contrast computed tomography and magnetic resonance imaging were diagnostic of an infarct involving the left frontal and parietal lobes. Carotid Doppler ultrasonography showed near-complete occlusion of the internal carotid artery. Tear film break-up time showed moderate dry eyes. Tests for rheumatological conditions showed a positive antinuclear antibody, and tests for extractable nuclear antigens showed positive anti-SS-A and anti-Ro-52. We diagnosed the case as a stroke due to clinically suspected Sjögren’s syndrome-associated large-vessel vasculitis and started the patient on prednisolone, azathioprine, and hydroxychloroquine, with a steroid taper over 4 weeks.

**Discussion:**

Postpartum stroke presents challenges in management because studies of thrombolytic therapy for stroke list pregnancy as an exclusion criterion. Our patient did not receive thrombolytic therapy; instead, she was managed with prednisolone, azathioprine, and hydroxychloroquine, with tapering of the steroid over 4 weeks.

**Conclusion:**

This case report highlights the challenges of postpartum stroke management and the roles of thorough investigations and interdisciplinary coordination.

## Introduction

Sjögren’s syndrome is a chronic systemic disease of rheumatic origin, characterized by lymphoplasmacytic infiltration of exocrine glands, primarily the salivary and lacrimal glands, characteristically giving rise to sicca (dryness) of the mouth and eyes^[^1^]^. It is considered primary when it occurs alone and is called secondary when it is associated with other autoimmune diseases, such as systemic lupus erythematosus (SLE), systemic sclerosis, primary biliary cirrhosis, thyroiditis, and vasculitis^[^1^]^.

HIGHLIGHTS

- Stroke is more frequent in pregnant than in non-pregnant young women, affecting 30 out of 100 000 pregnancies, with the highest risk in the peripartum period and the first 10 days postpartum.
- Rheumatic diseases, such as Sjögren’s syndrome, can flare in the postpartum period.
- Primary Sjögren’s syndrome can present with postpartum stroke.
- Interdisciplinary coordination between obstetricians and rheumatologists is needed for the early identification of postpartum flare-ups of rheumatic conditions and timely intervention.

Stroke occurs three times as frequently in pregnant compared with non-pregnant young adults and affects approximately 30 out of 100 000 pregnancies, with a higher risk in the peripartum and postpartum periods^[^2^]^. Hemodynamic, vascular, and connective tissue changes, as well as alterations in the coagulation system, predispose to the development of stroke, with an increased risk for 6−12 weeks postpartum^[^3^]^. The majority of readmissions for postpartum stroke occur within 10 days of discharge^[^4^]^.

We present a rare case of a postpartum woman presenting with a stroke, which was found to be large-vessel vasculitis due to clinically diagnosed Sjögren’s syndrome.

Our case report adheres to the CARE guidelines^[^5^]^.

## Presentation of the case

A 28-year-old woman presented to the emergency department 10 days postpartum with right-sided weakness of 27 hours duration, associated with deviation of the mouth toward the left side. The weakness was acute in onset, with tingling and heaviness of the right upper and lower limbs at onset, progressing to an inability to move the right upper and lower limbs completely. She also had slurred speech. No sensory disturbance was noted.

The patient was a multiparous woman (parity of two) who, following an uneventful term pregnancy with regular antenatal care, had a spontaneous home delivery of a 2.8 kg infant without complications.

On further inquiry after admission, she gave a history of generalized fatigability, aches and pains on and off, and dryness of the eyes for the past 5 years. She has no comorbidities.

On examination, her Glasgow Coma Scale score was 15/15, her vitals were within the normal range, and her face deviated to the left with loss of the nasolabial fold on the right side. An upper motor neuron-type facial palsy was observed. According to the Modified Medical Research Council grading of muscle power, it was 1/5 in the shoulder, elbow, wrist, hip, knee, and ankle on the right side, and 5/5 on the left side. Muscle tone was increased on the right side. On Babinski testing, the right side showed dorsiflexion of the big toe and fanning of the other toes (positive), while the left side showed plantar flexion (negative). Her National Institutes of Health Stroke Scalescore was 11 points.

Complete blood count, renal function tests, Prothrombin Time-International Normalized Ratio, and liver function tests were normal. Serology for Human Immunodeficiency Virus, Hepatitis C Virus, and syphilis was negative. Her electrocardiogram and chest X-ray were normal. Fasting lipid profile and thyroid function tests were normal.

Non-contrast computed tomography of the head showed a hypodense area in the left posterior parietal region suggestive of an infarct, with no midline shift or mass (Fig. 1). Magnetic resonance imaging (MRI) of the brain showed altered signal intensity in both gray and white matter of the left frontal and parietal lobes, left corona radiata, and left centrum semiovale, suggestive of an infarct.

**Figure 1.**
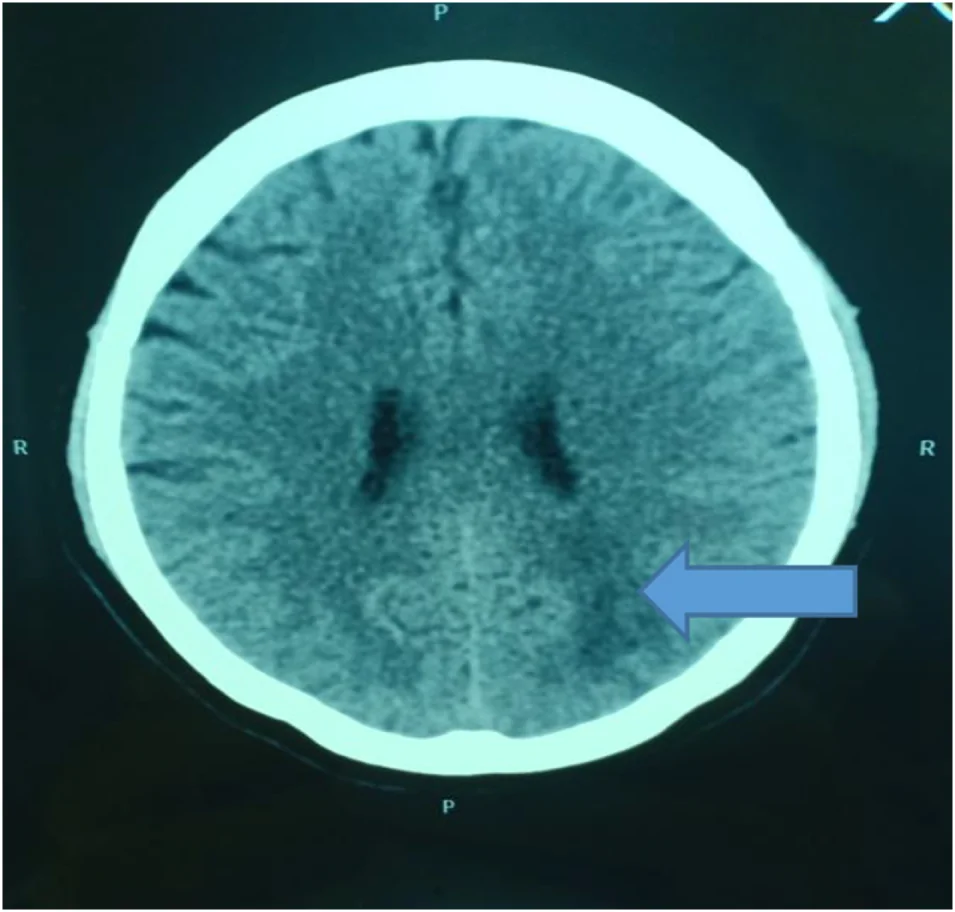
Non-contrast head CT, arrow showing a hypodense area in the left posterior parietal region at the level of the lateral ventricle.

Echocardiography revealed mild aortic regurgitation, normal left ventricular function, no thrombus or vegetations, and no septal defect.

A bilateral carotid Doppler was performed 4 days later, which showed an echogenic area in the left internal carotid artery causing near-complete occlusion.

Visual acuity was 6/9 in both eyes. Schirmer’s test: reflex, 35 mm in both eyes; basal, 18 mm in the right eye and 16 mm in the left eye. Tear film break-up time was 11.06 mm in the right eye and 9.04 mm in the left eye, with a diagnosis of moderate dry eye.

A 24-hour Holter ECG (24 h) showed no abnormalities.

Tests for anticardiolipin antibodies (IgG and IgM) and beta-2 glycoprotein I antibodies (IgG and IgM) were negative.

Tests for extractable nuclear antigens were positive for anti-SS-A and anti-Ro-52 (Table 1).

**Table 1:** Laboratory tests for extractable nuclear antigens, with SS-A and Ro-52 positive.

Extractable nuclear antigens	Result (intensity)	Interpretation
ds-DNA	0	Negative
Nucleosomes	8	Borderline positive
Histones	2	Negative
**SS-A**	**95**	**Strong positive**
**Ro-52**	**93**	**Strong positive**
SS-B	7	Borderline positive
nRNP/Sm	3	Negative
Sm	1	Negative
Mi-2α	1	Negative
Mi-2β	1	Negative
**Ku**	**29**	**Moderate positive**
CENP-B	0	Negative
CENP-A	1	Negative
Sp100	1	Negative
PML	1	Negative
Scl-70	2	Negative
PM-Scl100	1	Negative
PM-Scl75	5	Negative
**RP11**	**40**	**Moderate positive**
RP155	1	Negative
gp210	1	Negative
PCNA	1	Negative
DFS70	0	Negative
**Control (Co)**	**84**	**Strong positive**

Positive parameters are in bold.

The antinuclear antibody test was positive, showing a homogeneous nuclear pattern with multiphasic mitotic bodies. Intensity: 3+, initial dilution/titer: 1:80; endpoint dilution/titer: 1:320.

Due to the unavailability of magnetic resonance vessel wall imaging (MR-VWI) and financial constraints, further tests were not conducted. A salivary gland biopsy is another test for Sjögren’s syndrome, which was not performed because of a lack of patient consent. The patient has a score of 3 out of 9 in the 2016 American College of Rheumatology / European Alliance of Associations for Rheumatology classification criteria, which does not meet the required threshold of 4 or more for a Sjögren’s syndrome diagnosis, but, as most of the other tests, such as salivary gland biopsy and salivary flow rate, could not be done, we made a clinical diagnosis of Sjögren’s syndrome. As our case history and investigation panel were highly suggestive of Sjögren’s-related large-vessel vasculitis, we diagnosed this to be the cause of the stroke in our case.

## Management

Clopidogrel 75 mg and rosuvastatin 20 mg daily were initiated. She was kept in the intensive care unit for 4 days for monitoring. Aspirin was not administered because she was breastfeeding. A nasogastric tube was kept in place for 7 days for feeding, and supervised oral feeding was started thereafter. Physiotherapy was initiated early and continued.

The patient was managed in line with an ischemic cerebrovascular accident and was initially started on clopidogrel, and later, in view of antiphospholipid antibody (APLA) syndrome, it was changed to low-molecular-weight heparin and then to warfarin. When the APLA panel came back negative and further rheumatological tests suggested primary Sjögren’s syndrome and carotid Doppler revealed left internal carotid artery thrombosis causing complete occlusion, the antithrombotics were discontinued, and clopidogrel was restarted. The oral steroid prednisolone 60 mg, azathioprine, and hydroxychloroquine were also started. The patient continued breastfeeding, which was to be done 4 hours after the prednisolone intake. The steroid was tapered over 4 weeks. On discharge on the 10th day, power in the right half of the body had increased to 3/5. The sequence of events is presented in Table 2.

**Table 2:** Timeline showing the major events during hospital stay.

Time point	Event
Day 0	Uncomplicated spontaneous home delivery of a 2.8 kg infant.
Postpartum day 9	Acute onset right-sided weakness, facial deviation, and slurred speech.
Postpartum day 10	Presented to ER 27 h after symptom onset; NIHSS score 11. Examination consistent with left hemispheric stroke and right UMN facial palsy.
Admission day 1	Routine blood tests, ECG, CXR, lipid profile, TFT, and serology (HIV, HCV, and syphilis) normal.
Admission day 1	CT head: Left posterior parietal infarct.
Admission day 1	Started clopidogrel 75 mg and rosuvastatin 20 mg.
Admission day 2	MRI brain confirmed infarcts involving left frontal and parietal lobes, corona radiata, and centrum semiovale.
Admission day 4	Carotid Doppler: Near-complete occlusion of left internal carotid artery.
Admission day 5	Clopidogrel changed to LMWH, then warfarin due to suspected APLA syndrome.
Admission days 6-8	Ophthalmologic evaluation showed moderate dry eye disease. ANA, anti-SS-A, anti-Ro52 positive. APLA negative. Diagnosis made as Sjögren syndrome. LMWH stopped. Clopidogrel restarted; prednisolone, azathioprine, and hydroxychloroquine initiated.
Hospital day 10	Motor power improved to 3/5; discharged on medical therapy and physiotherapy.
Follow-up after 1 month	Improvement of muscle power to 4/5, no daily limitation of activities, no recurrences/complications.

CXR, Chest X-Ray; ECG, Electrocardiogram; ER, Emergency Department; HCV, Hepatitis C Virus; HIV, Human Immunodeficiency Virus; LMWH, Low Molecular Weight Heparin; NIHSS, National Institutes of Health Stroke Scale; TFT, Thyroid Function Test; UMN, Upper Motor Neuron.

The patient was followed up in the Out Patient Department after 1 month, and her muscle weakness in the right half of the body had significantly improved to 4/5, with no obstacles in performing daily household activities, and she had no recurrence of stroke or other complications.

## Discussion

In pregnant women, Sjögren’s syndrome can increase the risk of spontaneous abortion and cause preterm delivery, low birth weight, and birth defects in the fetus, such as heart block^[^6^]^. Postpartum flare is a common occurrence in rheumatic diseases, including Sjögren’s syndrome^[^7^]^. Sjögren’s syndrome has been reported to cause stroke in a few published case reports, but primary presentation as stroke in the postpartum period is extremely rare^[^8^]^.

Stroke in the postpartum period can have multiple causes. The absence of elevated blood pressure and of end-organ damage such as proteinuria, deranged organ function tests, and routine blood tests ruled out pre-eclampsia/eclampsia. No significant rhythm or structural abnormalities on ECG and echocardiography suggesting cardioembolism found in our patient. The possibility of antiphospholipid syndrome and SLE was also considered, but the patient did not fulfill the established diagnostic criteria for APLA (2022 ACR/EULAR criteria) or SLE (SLICC criteria).

Instead, our patient had persistent dry eyes, generalized aches and pains, and positive anti-SS-A and anti-Ro-52, which, in the absence of other causes, led us to diagnose the patient clinically with Sjögren’s syndrome with large-vessel vasculitis. The major limitations in this case include the absence of a salivary gland biopsy and MR-VWI.

Central Nervous System (CNS) manifestations in Sjögren’s syndrome are assumed to be due to immune-mediated vasculopathy resulting from autoimmune antibodies. Anti-Ro (SSA) antibodies are directly linked to the occurrence of vasculitis and CNS symptoms^[^9^]^.

Pregnant and postpartum women have similar favorable short-term outcomes following reperfusion therapy (thrombolytic, endovascular, or catheter-based). Little is known about thrombolytic therapies in pregnancy and the postpartum period because these are exclusion criteria in most reperfusion trials^[^10^]^.

## Conclusion

As postpartum patients are generally admitted to the obstetric ward, flare-ups of rheumatological conditions can be missed. In cesarean section patients, limb weakness might be misattributed to the effects of the surgery. Therefore, obstetricians should be well-versed in flare-ups of rheumatological conditions in pregnancy and should not miss the presentation of stroke. Overall, a presentation like this poses a diagnostic challenge, but careful observation and relevant investigations with interdisciplinary coordination can help diagnose uncommon presentations of rheumatic diseases such as Sjögren’s syndrome.

## Patient perspective

I was doing my household work when I suddenly felt unwell and developed weakness on the right side of my hand, which gradually spread to my legs. I rested for a while, but the weakness increased; I could not move the right half of my body, and my sister noticed that my face looked different too. I called my husband; we came to the hospital, and I was admitted. I underwent blood tests and other tests. It was difficult for me to breastfeed my child and even go to the bathroom. After hospitalization, my condition gradually improved, and after 1 month, I am grateful that I can do my daily activities without support and that I am getting better.

## Data Availability

Not relevant, as this is not a research study involving an interventional study in human participants or an observational study, but a report of a case.
